# The long road to routine care: piloting the digital mental health intervention for PTSD “Radius Grow” in a psychiatric residential setting

**DOI:** 10.1186/s40359-026-04738-5

**Published:** 2026-05-15

**Authors:** Sören Freerik Brähmer, Benjamin Iffland, Martina Bertino, Martin Driessen, Johanna Boettcher, Carolin Steuwe

**Affiliations:** 1 Clinical Psychology and Psychotherapy Working Group, Department of Psychology, Bielefeld University, Universitätsstraße 25, Bielefeld, 33615 Germany; 2 Department of Psychiatry and Psychotherapy, University Medical Center OWL, Evangelisches Klinikum Bethel, Bielefeld University, Remterweg 69-71, Bielefeld, 33617 Germany; 3 Clinical Psychology and Psychotherapy Working Group, Psychologische Hochschule Berlin, Am Köllnischen Park 2, Berlin, 10179 Germany

**Keywords:** Digital mental health, Blended Care, Post-traumatic stress disorder, Residential care, Cognitive behavior therapy, Implementation, Pilot study

## Abstract

**Background:**

While psychotherapeutic interventions for Post Traumatic Stress Disorder (PTSD) are effective, transfer to everyday life is challenged by a shift from treatment setting to the patients’ living environment. Adding digital components to the treatment (Blended Care, BC) may support autonomous completion of therapeutic tasks and facilitate transfer but has not been studied in residential treatment settings.

**Objective:**

The present study explores the implementation, usability, and preliminary effectiveness of a BC approach for PTSD treatment in a non-acute residential hospital setting. Clinician and patient perspectives are considered.

**Methods:**

36 patients participated in a two-group quasi-experimental design (intervention vs. control) with three measurements (admission, discharge, three-month follow-up). Face-to-face trauma-focused CBT was offered in both groups, complemented by a BC software in the intervention group. Also, 16 clinicians participated in the study. We collected qualitative data from semi-structured interviews, software usage-data, and psychometric questionnaire data from patients and clinicians.

**Results:**

Patients reported high satisfaction with software usability; clinicians had varying opinions. Uptake was inconsistent on both sides. Capacity issues and an inaccurate definition of the intended use hampered implementation. Still, intensified collaboration and sustained engagement with treatment contents were identified as benefits of BC. Quantitative measures of symptom severity showed no significant between-group differences in exploratory analyses and no relation to software usage intensity.

**Conclusions:**

This study confirms potentials and obstacles of BC to facilitate the therapeutic process in the residential treatment of PTSD. It also emphasizes the importance of a concise concept for the coordination of digital and face-to-face components to ensure sufficient uptake and raise clinical effectiveness.

**Trial registration:**

drks.de, DRKS00031741, Registration date: 21 April 2023, https://drks.de/search/de/trial/DRKS00031741/details.

**Supplementary Information:**

The online version contains supplementary material available at 10.1186/s40359-026-04738-5.

## Theoretical background

### Recurrence of PTSD in residential treatment

Post-traumatic stress disorder (PTSD) affects 1.3–8.8% of the global population [1, 2] and takes a chronic or recurrent course in almost 40% of the cases [3, 4] . Also, a high rate of non-response of almost 40% is an ongoing problem in PTSD-treatment [5] . With respect to learning theories, the recurrence of PTSD after treatment is due to the reuptake of avoidance behavior or experiential avoidance, initiated by a change of context and stabilized by negative reinforcement [6, 7] . Residential treatment is particularly vulnerable to this relapse mechanism for two interrelated reasons. The first relates to the target population. Patients admitted to non-acute psychiatric residential treatment (for PTSD and other disorders) typically present with a severe and complex course of disease [8, 9] . Also, they are often at high risk of a crisis [10] . These patients are likely to have a history of unsuccessful therapeutic interventions and a high, acute burden of disease, which complicates the acquisition and application of new cognitive and behavioral strategies. The second hurdle for successful everyday transfer is closely linked to the structure of non-acute residential treatment. This treatment format is characterized by intensive, multimodal programs and shielding patients from the stressors of everyday life [9, 10] . However, being shielded from everyday life makes it difficult to transfer therapy content from the treatment environment (e.g., the residential ward) to a person’s domestic environment (e.g., their home) [11] . Although there is no evidence linking these specific mechanisms causally to heightened relapse rates, residential treatment of PTSD is often associated with high pretreatment symptom scores and high relapse rates [12, 13] .

### Blended care in PTSD treatment

Bridging the gap between therapy and everyday life is an often-quoted promise of Blended Care (BC), which denotes the combination of digital and face-to-face therapy [14] . The digital component is designed to make therapy contents more accessible in everyday life (e.g., through accessible documentation or interactive material for psychoeducation) while the face-to-face component should ensure treatment acceptability and individual tailoring [15] . Although most research on BC has targeted outpatient treatments [16] , a growing body of literature underlines the potential of BC to increase the efficacy of residential care [17] . Digital components have been found useful both as an adjunct [18] and as a follow up [19] to residential treatments.

Few specific BC approaches have been tested for PTSD. The PE Coach application provides psychoeducation and homework assignments that accompany manualized prolonged exposure therapy [20] . Homework completion rates were higher for sessions with the software than without. While large symptom reductions were reported post-treatment, these were not compared to a control group. The MORPHEUS program [21] , based on Dialectical Behavior Therapy and examined in a PTSD sample, enables self-managed imaginative exposure through playback of recorded therapist guided exposure sessions. During self-managed exposure, it facilitates monitoring of state dissociation and offers interactive distraction skills. The use of distraction skills was associated with decreased dissociation but treatment effects on PTSD severity have not been reported in the study. The available evidence suggests that BC may add structure to therapeutic processes and support patients in the self-sustained realization of therapeutic tasks such as exposure and application of stabilizing techniques [22] . Thus, BC may facilitate transfer to everyday life which could lead to more sustainable effects in the treatment of chronic and recurrent PTSD. This applies to all therapy settings, but it seems particularly valuable in residential settings.

### Implementation of blended care in residential settings

A central challenge in previous studies on BC in residential settings has been low uptake [18, 23, 24, 21] . In fact, the MORPHEUS skills were used only by a subset of patients and, on average, four times over a 12-week residential treatment [21] . Specific implementation barriers in residential settings have not yet been examined consistently. Nevertheless, the literature provides some hypotheses on implementation determinants. For example, usage of the MORPHEUS application was not supervised by the treating clinicians and patients wished for more support. In comparison, usage of the PE Coach application (outpatient setting) was supervised by the treating clinician. Patients reported higher satisfaction with sessions supported by the PE Coach than without [20] . These results indicate that guidance by clinicians may be important for patients’ usage behavior of BC tools for PTSD. A similar suggestion has been made regarding BC for depression in a residential setting [25] and BC in outpatient settings [26] . Patients’ usage behavior thus seems to be influenced by clinicians’ engagement with BC tools, across disorders and settings. Professionals often criticize a lack of conceptual integration between digital and face-to-face treatment components [27] . Nevertheless, they tend to prefer BC to standalone digital interventions [28] . From studies in outpatient settings, it can further be concluded that cooperation and communication between clinicians and patients can be facilitated with digital tools. Helpful elements are the reduction of organizational barriers through asynchronous communication, more constant availability and a lowered threshold for seeking contact e.g. due to reduced shame [22, 29] . This may potentially benefit working alliance [30] . On the other hand, clinicians frequently express concerns about a potential neglect of face-to-face contacts [27] .

Overall, implementation remains “the next giant hurdle” in the residential application of BC [31] . Previous results highlight the importance of clinician engagement, yet professionals often have mixed feelings about BC. Furthermore, no study to date has investigated whether applying BC approaches can actually improve the outcomes of residential treatment of PTSD. This is important, however, since the perceived added value has been identified as a key determinant of the implementation of digital mental health tools [32] . It therefore seems timely to evaluate how BC can be integrated with existing processes in the residential treatment of PTSD and how this interacts with the guidance provided by clinicians, with the working alliance between clinicians and patients, and with the perceived utility of BC.

### Study rationale

This study is part of the EU-funded project “IT4Anxiety”, which aimed at the co-development, testing, and implementation of technological advancements in the care of persons suffering from anxiety. The project mission was to connect patients, clinicians, researchers, and developers. The present study reports on a pilot trial of a BC-software (Radius Grow) in a real-world non-acute hospital setting, designed to accompany and sustain the treatment of PTSD. Our primary aim was to explore the implementation process, determinants of acceptance of the BC approach and the way it was used under naturalistic conditions. We were interested in the perspectives of clinicians as well as patients. Based on the literature presented above, we assumed that guidance and working alliance would be important determinants of uptake, but that the communication between clinicians and patients might also be influenced (either facilitated or hindered) by the introduction of the digital tool. We also assessed software usability in terms of user experience and efficiency of use (following DIN ISO 9241 [33] ) and assumed that it would be sufficient for routine implementation. As a secondary aim, we strove to generate preliminary estimates of clinical effectiveness by including quantitative measures of symptom severity and working alliance into an exploratory analysis.

## Methods

### Setting

Recruitment took place in a German psychiatric hospital on two wards specialized in the treatment of PTSD for patients with a severe and long course of disease or complex comorbidities. This non-acute hospital setting covers psychotherapy in weekly individual and group sessions (typically one individual session with a psychologist and one with a psychiatric nurse per week, and additional group sessions), psychiatric medication, adjuvant therapies (e.g., occupational therapy, art therapy) and social work. Trauma-focused therapy in this setting is focused on cognitive behavior therapy using psychoeducation, stabilization methods, and exposure therapy (in-vivo or in-sensu). The standardization of treatment components and the order in which they were provided is precluded by the naturalistic design. Treatments usually last one to three months.

### Design

We chose a two-group quasi-experimental design with three measurements (admission, discharge, three-month follow-up). We collected qualitative and quantitative data. Treatment allocation was not randomized, instead we recruited the control group first and the intervention group second. Due to the residential setting, informal exchange between the groups might otherwise have confounded patients’ feedback. Recruitment and assessment took place from April 2023 until September 2024. In both groups, face-to-face trauma-focused CBT was offered. In the intervention group, the Radius-Grow software was offered as an adjunct. It was available during residential treatment and for three months after discharge. Due to the design of the study groups, blinding was not possible for any participants. The study protocol adhered to the Consolidated Standards of Reporting Trials (CONSORT) statement [34] and the extension for pilot trials [35] .

### Sample size

In line with the recommendations of the extended CONSORT statement for pilot trials [35] , we adjusted our sample size according to the scope of our study, which falls between software development and large-scale effectiveness testing. According to Guest et al. [36] , 92% data saturation in qualitative data analysis is reached after ten interviews. Regarding quantitative data analysis, similar pilot trials evaluating usability and uptake used samples of 15 to 30 patients [23, 18, 29, 21, 20] . To our knowledge, no estimates for the effects of residential BC on PTSD symptom severity are available to date. We thus chose a target sample size of 15 patients per group (total *n* = 30). This is adequate for the analysis of qualitative data, quantitative uptake measures and the exploratory analysis of clinical effects.

### Participants

The sample consisted of patients and the clinicians involved in their treatment (psychiatric nurses, psychologists). Inclusion criteria for the patients were a diagnosis of PTSD according to ICD-11, a treatment duration of four weeks or longer, age between 18 and 65 years and availability of an internet-enabled device. Exclusion criteria were acquired brain damage, CNS-related somatic diseases, serious somatic illness requiring urgent treatment, current life-threatening crises with acute suicidality, psychotic disorders (current or within the last year) and eating disorders with BMI < 15. Clinicians had to be psychologists with clinical training, psychiatrists or psychiatric nurses to be included in the study, and they had to be involved in delivering trauma-focused therapy. In total, 36 patients fulfilled the eligibility criteria and gave informed consent (intervention group: *n* = 21, control group: *n* = 15). Study dropout was similar in both groups, with 19 patients (91%) in the intervention group completing questionnaires at discharge and 13 (62%) at follow-up (control group: 13 (87%) at discharge and 11 (73%) at follow-up). In the intervention group, reasons for dropout included premature end of treatment (private reasons), crisis situations and substance abuse relapse after discharge, acute somatic condition, refusal to continue participation after discharge (too busy) and perception of the questionnaires as too strenuous or intimate. In the control group, reasons for dropout were similar and included crisis situations after discharge, perception of the questionnaires as too demanding in terms of concentration or stress, and non-reaction to attempted contact. The mean age of patients was m = 39.49 years (sd = 13.34) and 61% (*n* = 22) identified as female. 88.9% had at least one comorbid ICD-11 mental disorder, the most common being major depressive disorder (*n* = 18), substance abuse disorders (*n* = 18) and personality disorders (*n* = 7). The mean treatment duration was m = 44.97 days (sd = 11.31). 16 clinical staff members participated in the study (seven psychologists, nine psychiatric nurses). Mean age was m = 39.0 years (sd = 10.99) and 69% (*n* = 11) identified as female. All 19 intervention-group patients who participated at discharge also participated in the qualitative interviews. Four clinicians were involved only in the control group, leaving twelve clinicians who participated in the qualitative interviews. This sample size was considered sufficient to gather most of the relevant information.

### Study procedure

All clinicians provided informed consent and received training on the use of Radius-Grow. Subsequently, all newly admitted patients seeking treatment for PTSD were approached by the research personnel and informed consent was obtained. The diagnosis of PTSD was asserted during admission to the hospital and corroborated by the International Trauma Questionnaire (ITQ) [37] as part of the study procedure. One reference clinician was identified for each patient, who reported on the working alliance (see Sect.  2.6.) and supervised the usage of Radius-Grow. For each patient in the intervention group, an introduction to Radius-Grow was given by the reference clinician and a researcher. Technical support was offered by the developer of the software (Circumradius GmbH, Stadthagen, Germany). Assessments were scheduled for the day of admission (the first measurement) and the day of discharge (the second measurement). For practical reasons, an additional +/- 2 days were allowed. For the three-month follow-up, a link to a digital questionnaire was sent one week before the end of the period, requesting completion within one week. Reminders were sent via email on the follow-up date and via telephone one week later. Responses entered more than two weeks after the assigned follow-up date were not considered.

### Intervention

In line with the IT4Anxiety project mission, the development of the web-app “Radius Grow” (Circumradius GmbH, Stadthagen, Germany) incorporated input by patients and clinicians through three focus-groups and a joint workshop. The version of the software used in this study could be accessed through any browser on any internet-enabled device. It was designed to complement, not to replace face-to-face treatment components. It aimed at overcoming avoidance behavior and making therapeutic content readily available, thereby grounding new behavioral strategies in everyday life. The software could be handled by the patient, by the reference clinician, and by up to one additional clinician. Patients and clinicians had their own interfaces.

**Fig. 1:**
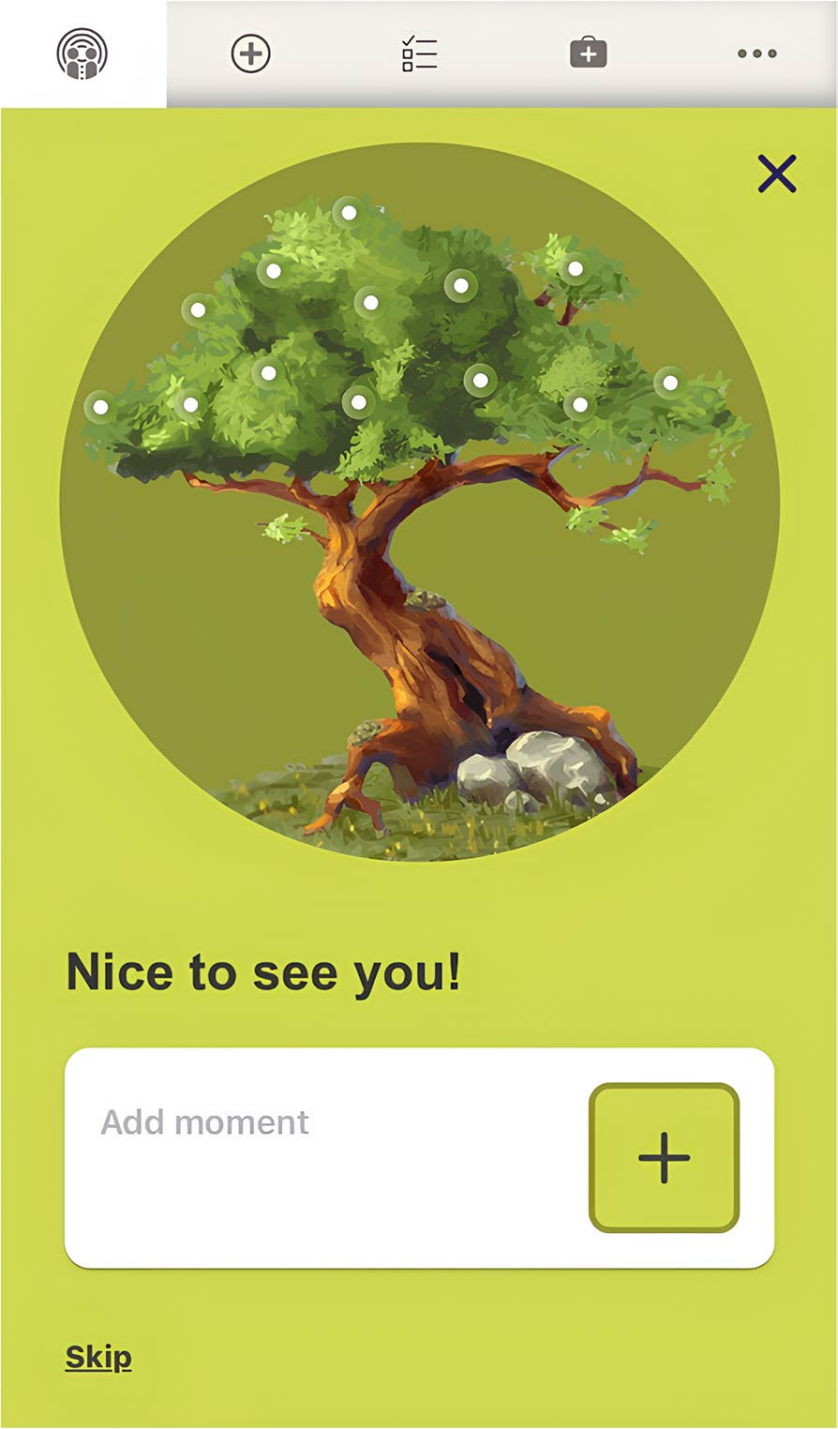
Sample screen from the “Radius-Grow” patient interface

Goals could be set and scheduled (by patients and clinicians) with a reminder function. To encourage the reduction of avoidance behavior, a gamified logbook was included (Fig. 1). Overcoming avoidant behavior during exposure tasks or everyday life was documented by the patient by a text entry, a picture and/or emoticons (so called “moments”). The clinician could give feedback via an integrated messenger. The combined use of (recurrent) goals and the collection of “moments” was meant to procure a lasting repository of coping experiences and to build up new behavioral routines. This was inspired by the “reclaiming your live” module of trauma-focused CBT [38] . To further support the treatment, the course of symptoms could be tracked by customizable questionnaires, the results of which were presented in progression charts. The software also allowed documentation of skills and emergency contacts. There were no precise requirements regarding usage intensity. Communication between patients and clinicians remained possible after discharge, if desired by both parties.

### Data collection & measures

Patients and clinicians who were involved in the intervention group participated in semi-structured qualitative interviews. Interviews with patients were conducted at discharge, interviews with clinicians were conducted after completion of all treatments. The interview guides were based on the DIN ISO 9241 model of usability [33] , the Normalization Process Theory (NPT) [39] and the Consolidated Framework for Implementation Research (CFIR) [40] . They focused on usability and the implementation process (example question clinicians: To what extent is Radius-Grow useful for your work? Example question patients: How did Radius-Grow affect your treatment? See supplementary material A for the full interview guides). Also, negative effects were systematically assessed by the interviews. The interview guides were cooperatively developed by the research team. All interviews were conducted by S.B. on the hospital premises and lasted around 20 min. The interviews were tape-recorded, transcribed verbatim with the aid of artificial intelligence [41] and checked against the original recording in full length.

To quantify program uptake, user data (logins, activities) for patients and clinicians was collected directly from the software. Regarding quantitative measures of usability, symptomatology and working alliance, patients and clinicians also completed psychometric questionnaires on several occasions (Table 1). The questionnaires on symptomatology also allow to test for deterioration, which is a common measure for harmful effects [42] .

**Table 1:** Psychometric measures

Construct	Instrument	Details	Measurement		
Patients			Admission	Discharge	3-month follow-up
Usability	SUS^1^ [43]	Sum Score Range: 0-100; Score > 74 denotes good usability [44]		X^8^	
Acceptance	CSQ-3^2^ [45]	Mean Range: 1–4		X^8^	
PTSD symptoms	ITQ^3^ [37]	Sum Score Range: 0–24; Reference value for traumatized persons: 15.84	X	X	X
Avoidance behavior	PABQ^4^ [46]	Sum Score Range: 25–100; Reference value for PTSD patients: 57.6	X	X	X
Symptoms of depression	BDI-V^5^ [47]	Sum Score Range: 0-100; 90% probability of clinical relevance for scores ≥ 35	X	X	X
Working alliance	WAI-SR-P^6^ [48]	Mean Range: 1–5	X	X	
Clinicians			Admission	Discharge	End of study
Usability	SUS^1^	Sum Score Range: 0-100			X^8^
Working alliance	WAI-SR-T^7^ [48]	Mean Range: 1–5	X	X	

^1^System Usability Scale ^2^Client Satisfaction Questionnaire ^3^International Trauma Questionnaire ^4^Posttraumatic Avoidance Behavior Questionnaire ^5^Beck Depression Inventory V ^6^Working Alliance Inventory-Short-Revised-Patient Version ^7^Working Alliance Inventory-Short-Revised-Therapist Version ^8^Only in intervention group

The measures of usability, acceptance and working alliance were most relevant to the primary objective of the study (implementation and feasibility). Measuring avoidance behavior was related to the proposed mechanism of the intervention. Symptoms of PTSD and depression were the clinical outcomes targeted by the treatment as a whole.

### Qualitative data analysis of semi-structured interviews

Given the pre-existing literature on implementation science, especially the Consolidated Framework for Implementation Research [40] , we chose a combined deductive-inductive, realist approach. Deductive codes were informed by the constructs of the updated CFIR [49] . We then followed the process of Framework Analysis [50] : after transcription of the interviews (step 1) and familiarization with the data (step 2), inductive codes were identified from field notes and during initial coding (by discussion among authors S.B., C.S., and B.I.) (step 3: coding). After the research team settled on a preliminary thematic framework, indexing of the complete dataset was done by S.B. (step 4: indexing). During this process, the thematic framework was repeatedly adapted. The final thematic framework was discussed and agreed on by the whole research team, leading to the identification of central themes, which are presented below (step 5: charting and interpretation). Quotations were translated to English by the authors.

### Exploratory quantitative data analysis of psychometric questionnaires

Descriptive analyses of usage data and usability are presented. For the System Usability Scale, one patient had to be removed for not filling out the questionnaire. Otherwise, there were no missing values on the SUS. With regard to our secondary purpose (clinical effectiveness estimates), psychometric questionnaires on symptoms and working alliance were analyzed using linear mixed models (LMM). LMMs avoid listwise deletion by using all available observations at each measurement (i.e., if a participant misses a questionnaire score at one measurement, their scores from the remaining measurements can still be analyzed). Within completed measurements, missing item values were replaced by the person means at the respective measurement (missing items CSQ = 2.5%, ITQ = 1.1%, PABQ = 1.5%, BDI-V = 1.2%, WAI-SR-*P* = 3.7%, WAI-SR-T = 0.3%). Repeated measures were modeled by nesting measurements (admission, discharge, follow-up) in patients (i.e., level 2). Patients were nested in clinicians (i.e., level 3). Random intercepts were allowed on levels 2 and 3. First, we used group (intervention vs. control) and its interaction with measurement to predict questionnaire scores. Effect sizes were calculated using the difference in estimated means between admission and discharge/follow-up values (within-group) or between the group means at one measurement (between-group). Following Westfall et al. [51] , these values were standardized by using the pooled standard deviation of the respective model, using all variance components. The resulting effect sizes correspond to Hedges’ d (generalized) [52] . To accommodate the smaller sample size, we transformed Hedges’ d to Hedges’ g, which is commonly categorized as g > 0.2 = small effect, g > 0.5 = medium effect and g > 0.8 = large effect [53] . Given the heterogeneous uptake of the intervention (see Sect.  3.3.), we added a supplementary analysis, using the usage intensity (login rate) as a continuous predictor for the symptom change scores (i.e., dose-response relationship). Standardized regression coefficients from the LMMs were used as effect sizes. All model specifications can be found along with the detailed results in supplementary material B. All computations were performed with R Version 4.4.2 [54] .

## Results

### Clinician interviews

For the clinician interviews, we summarized the results from Framework Analysis into four themes. Code frequencies are shown in Table 2.

**Table 2:** Codes and frequencies of clinician interview analysis

Theme	Code	Participants *N* [%]	Frequency
Benefits and limitations of Radius-Grow	Added value	11 [92%]	44
	Absent / negative effects	6 [50%]	8
	Conceptual integration & compatibility	11 [92%]	19
	Suggestions for improvements to app	7 [58%]	13
	Concerns about added workload	6 [50%]	16
	Concerns about added liability	8 [67%]	16
	Team processes	11 [92%]	49
Usability	Positive usability aspects	8 [67%]	16
	Negative usability aspects	10 [83%]	21
	Own IT-competencies	3 [25%]	3
Routine integration	Routines and motivation	11 [92%]	31
	Own usage behavior	10 [83%]	26
Interacting with patients	Motivational work / support for patients	10 [83%]	24
	Patients’ motivational barriers	9 [75%]	14
	Patients’ usage behavior	9 [75%]	27

The first theme we labelled “benefits and limitations of Radius-Grow”. It focuses on the added value of Radius-Grow and its conceptual integration into the treatment. Most clinicians saw Radius-Grow mainly as a tool for relapse prevention: staying in touch after discharge was seen as an important added value, not only for the patients but also to learn about positive developments (“*I also showed the photo of Mr. W. because it was simply beautiful. Relaxed in front of the sea. […] We often see the ones who keep coming back*,* where you work and only move at a snail’s pace for a long time*.”). On the other hand, this provoked uncertainties regarding added workload and responsibilities in case of deterioration. Less frequently, Radius-Grow was mentioned as a sensible supplement during residential treatment (e.g. to facilitate goal setting or to share a record of agreements with patients). A potential future exploit was seen in establishing mutual feedback between patients to reinforce their progress (e.g. via comments or reactions to entries). Regarding the interaction among professionals, some saw the software as *“[…] redundant on the ward because you already have most of the important information*”. Others thought that the tool led to closer cooperation between different professions and thus more appreciation of other’s work.

The second theme focused on “usability”. Clinicians had varying opinions on the design and the user experience. The design was seen in turns as “*sober*”, “*unattractive*”, or “*totally okay*”. A frequent critique resembled that “*the overview in Radius-Grow just wasn’t that good*” which slowed down work processes. Only very few remarks addressed a general lack of IT competencies as a hurdle.

Difficulties with the “routine integration” of the software were collected in the third theme. They resulted from frequent disruptions in the working process, forgetting about Radius-Grow or deliberately assigning a low priority to it. Others set up straightforward routines by linking the usage of Radius-Grow to certain weekdays or team meetings. One participant concluded: “*If you want to implement this*,* then you can only do so if it is firmly established somewhere in the structure of the day or week*.”

The heterogeneous conceptual and practical integration translates to the amount of guidance given to patients, which we subsumed under the fourth theme, “interacting with patients”. While some clinicians proactively provided structure and reminders (“*So while they are on the ward*,* yes*,* I actively follow them up and try to help them internalize that a little bit into their everyday life.*”), others took a more neutral stance, conditioning their level of commitment on that of the patients. Overall, the uptake by patients was felt to be slow. This was primarily attributed to shyness about self-disclosure, avoidance, and lack of automated reminders.

### Patient interviews

Contents from the patient interviews were categorized into three themes. Code frequencies are shown in Table 3.

**Table 3:** Codes and frequencies of patient interview analysis

Theme	Code	Participants *N* [%]	Frequency
Acceptance and usability	Positive attitudes	11 [58%]	19
	Negative attitudes	6 [32%]	8
	Positive usability aspects	16 [84%]	40
	Negative usability aspects	10 [53%]	24
	Suggestions for improvements to app	9 [47%]	24
	Own IT-competencies	4 [21%]	7
	Technical issues & support	10 [53%]	24
Preliminary positive and negative effects	Added value during treatment	17 [90%]	46
	Added value after treatment	11 [58%]	29
	Absent / negative effects	11 [58%]	18
Conceptual understanding and routine integration	Conceptual understanding	16 [84%]	42
	Usage intensity	17 [90%]	48
	Capacity / time issues	12 [63%]	33
	Clinicians’ usage behavior	18 [95%]	46

The first theme, “acceptance and usability”, includes remarks about positive and negative attitudes towards the BC, as well as comments on usability. Most patients expressed a positive attitude towards the blended concept. In several cases, the documentation of skills and emergency contacts was felt to be obsolete since better solutions already were in place. With some exceptions, patients felt sufficiently competent to handle the software and stated that it was clearly arranged. Criticism was expressed regarding the process of accessing the web-app, the lack of push-notification reminders and a missing possibility to modify or delete “moments”.

The second theme concerned the “preliminary positive and negative effects” of the Blended Treatment. Regarding negative effects, software malfunctions led to different levels of frustration. One patient reported to have written about his exposure therapy in the evenings, leading to discomfort: “*Then what I wanted to write came up again*,* which was just a bit awkward.*” Two patients feared that they would be obliged to talk about their entries. In contrast, lowering the threshold for getting in touch with staff was seen as a central benefit by most others. This concerns overcoming reluctance against asking for help (“*I used to be in the background*,* more or less*,* and didn’t dare to talk to anyone*,* and this helped a lot*”) or a preference for non-verbal content (i.e., pictures, emoticons). Asynchronous communication also had the very pragmatic advantage of always addressing the right person. Some patients reported that clinicians read their entries before therapy sessions, making it possible to use the session more efficiently. Independent from clinician usage, the software helped to cope with bad mood by retrieving positive memories, looking at positive developments in the symptom course and accessing coping strategies. Regarding the time after discharge, some emphasized the option to get in touch on-demand, resulting in a feeling of security. Others valued the possibility to write about their feelings (“*You get it off your chest*”), combined with individualized feedback from the treating clinician. Others again focused on keeping therapy contents and achievements present instead of being superseded by everyday life.

More controversial than the handling and the utility of the software was its “conceptual understanding and routine integration”, which was the third theme. It seemed important that patients found a meaningful way to use Radius-Grow and an appropriate frequency of use, rather than using it as often as possible (“*But it actually became such a regular part of my week that I really take time on Thursdays to gather all my thoughts together and then really concentrate on them*”). In this regard, patients appreciated reliable feedback on their entries. On the other hand, when they did not receive feedback, patients stated that they did not know what kind of content or what frequency of use was expected of them. In this case, several patients used the software as a (positive) diary. Some also abandoned the software. During their residential care, some patients received trauma-specific psychoeducation and stabilization rather than exposure therapy. Among those who did receive exposure, only a few used Radius-Grow for setting avoidance specific goals and documenting reduction of avoidance behavior. Those who did found it helpful for this purpose.

Even when patients knew what they wanted to use Radius-Grow for, they often reported not having the capacity for it. In this regard, the therapy was described as effortful (“*And because of my exposure therapy*,* I was of course also a bit*,* often lacking the concentration to get it right”)*. In addition, practical concerns (e.g., housing, finances) demanded cognitive capacity. Difficulties structuring oneself or taking time for oneself were reported as further barriers to frequent usage.

### Usability and usage data

On average, patients had access to the online platform on 118.4 days (sd = 17.4) (from creating the account during treatment until shutting it off three months after discharge). They logged in on m = 15.3 days (sd = 16.6), i.e. the average log-in rate was 12.6% (sd = 12.9%, range = 0–51%, median = 8%). This includes two patients who never used their accounts after creation. Goals were used by 19/21 patients (m = 8.7 goals, sd = 6.9, range = 2–24) on m = 2.6 days (sd = 1.5). The collection of “moments” was used by 13/21 patients who collected on average 12.5 moments (sd = 9.5, range = 1–31) on m = 8.2 days (sd = 5.8). Messages were sent by 10/21 patients (m = 6.9 messages, sd = 6.4, range = 1–16) on m = 5.9 days (sd = 5.2). At discharge, the patients’ usability score (SUS) for Radius-Grow was m = 81.25 (sd = 9.52, range = 60.0-97.5), which according to Sauro and Lewis [44] denotes good usability. The acceptance score (CSQ m = 3.41, sd = 0.68, range = 2–4) indicated that on average patients were satisfied with Radius-Grow.

Clinicians had access on m = 324.86 (sd = 45.96) days. They logged in on 3–32% of the available days (m = 17.4%, sd = 13.1%, median = 12%). The usability ratings by clinicians showed high variability but were overall lower than those of patients (SUS m = 68.1, sd = 20.4, range = 30.0-97.5).

### Symptoms and working alliance

A small to medium treatment effect could be observed for the severity of PTSD (ITQ) both in the intervention group (g_discharge_=-0.65, g_follow−up_=-0.64) and the control group (g_discharge_=-0.51, g_follow−up_=-0.46). Similar results were obtained for depressive symptoms (BDI-V) (intervention group: g_discharge_=-0.58, g_follow−up_=-0.31; control group: g_discharge_=-0.45, g_follow−up_=-0.22). These values are reflected in significant effects of the measurement on both ITQ-values (F(2, 56.05) = 6.65, *p*=.003) and BDI-V-values (F(2, 56.13) = 3.87, *p*=.027). Avoidance behavior (PABQ) stayed roughly at the same level across all three measurements. For none of these outcomes, the course of symptoms differed significantly between groups (i.e., no significant main effect of group, no significant interaction of measurement and group). Descriptively, patients in the intervention group reported a small improvement in the working alliance (WAI-SR-P) (g_discharge_=0.43) which was not observed in the control group (g_discharge_=-0.03). Clinicians didn’t report any differences in working alliance between groups (WAI-SR-T). None of the effects on working alliance reached statistical significance. The estimated means and effect sizes from the multilevel models with the variable “group” as a predictor are presented in supplementary material B (supplementary table S1). The model specifications and results can be found in supplementary tables S2-S6. To account for the varying uptake, we used the usage intensity (the login rate = proportion of days with at least one login) as a predictor of the symptom change scores (i.e., the difference between admission and discharge, and admission and follow up). Due to the quasi-experimental design, it was not possible to fully rule out cohort effects based on unobserved confounding variables. Therefore, this supplementary analysis only used data from the intervention-group data. There was no significant effect of usage intensity on any outcome measure, neither at admission nor at discharge or at follow-up (supplementary table S7). The model specifications and results can be found in supplementary tables S8-S12.

## Discussion

This study’s objective was the piloting and preliminary implementation of a Blended Care (BC) program for Post Traumatic Stress Disorder (PTSD) in a routine care residential setting. Our primary objective was to identify implementation determinants and assess feasibility (i.e., uptake and usability) in a naturalistic setting. To this end, we chose a combined qualitative-quantitative approach incorporating the views of patients and clinicians. Our secondary objective was to explore clinical effectiveness by comparing the symptom course between a BC-group and a treatment-as-usual (TAU) group.

### Implementation and feasibility

Barriers to routine uptake among clinicians included workflow management, cognitive overload and limited time. While some criticism was expressed about the usability of the software, it was rated as sufficient on average. Although Radius-Grow was developed specifically to support trauma-focused therapy, clinicians had different views on how it was conceptually connected to their work. This may be due to differences in professional background and experience (e.g., nurses vs. psychologists, psychologists with vs. without specialization in trauma-focused therapy), although no clear pattern emerged from the qualitative analysis in this regard. Accordingly, even in the absence of other barriers, clinicians took varying degrees of responsibility for motivating patients to use the software. Patients reported that the software was easy to use but criticized the process of accessing it and the absence of push notifications. Like the clinicians, some patients indicated that limited time or low priority undermined routine use.

In qualitative reports of added values, facilitated communication between patients and clinicians (during and after treatment) was the most frequently mentioned benefit in the interviews with both user groups. This may be supported by the quantitative results which show a trend towards improved patient-rated working alliance in the intervention group. Patients also found the software helpful for saving positive memories and relevant therapy content, accessing coping strategies, and reported that it facilitated preparation, making therapy sessions more efficient. About half of the patients mentioned negative or absent effects of Radius-Grow, mostly referring to technical issues but also to discomfort after writing about feelings, and fear of exceeding self-disclosure.

It became clear that users’ evaluation of the BC program as helpful or not is only one () determinant of its uptake, albeit an important one. This is consistent with implementation theories [40, 55] . In terms of the Consolidated Framework for Implementation Research (CFIR), personal capabilities and the compatibility with existing processes were important implementation determinants in this case (also called “coherence” in Normalization Process Theory (NPT) [39] ). Although the flexibility of the tool enabled it to be adapted to existing processes, this also required time and cognitive capacity (“cognitive participation” in NPT) which may have been overwhelming in this residential treatment setting.

These factors may have resulted in a discrepancy between the positive evaluation of the program evaluation and its inconsistent uptake. Here, 19 out of 21 patients (91%) used the software, with an average log-in rate of 12.6%. Previous studies on BC in residential settings, too, report a discrepancy between high usability and low usage. Regarding PTSD treatment, Görg et al. [21] tested the MORPHEUS program, which was used 2–5 times per week. However, only 64% of participants used the skills built into the software. Sharma et al. [23] reported study completion rather than actual usage of their CBT app, which targeted anxiety symptoms. Low study completion (55%) was attributable to the residential treatment setting rather than the tested software, but this still demonstrates that achieving complete intervention uptake is difficult in such settings. Similarly, Bendig et al. [18] found that 60% of participants completed all modules of their digital social skills training, while Wälchli et al. [24] found that only 12 out of 30 patients (40%) completed at least 50% of their digital emotion regulation intervention. Although using different uptake measures hinders a direct comparison, it is reasonable to assume that the uptake of the software in the current study is consistent with the relevant literature on this topic. Also, the average usage rate of 12.6% corresponds to approximately one usage per week. In the residential setting in which this study took place, many psychological treatment components are provided once weekly. Therefore, usage of Radius-Grow is comparable to that of other treatment components. Importantly, patients who managed to set up regular (not necessarily highly frequent) usage of Radius-Grow were satisfied with their own uptake of the program.

In line with the previous studies, patients reported that reliable guidance was helpful in committing to routine use [26] . In order to provide consistent guidance, clinicians themselves need to develop a shared understanding of how to integrate the digital tool with the TAU and their everyday work. Standardizing workflows and treatment procedures is a challenge for any (psychological) intervention to be implemented in residential settings [56] . Therefore, implementing new procedures in healthcare settings is a lengthy process that requires the therapeutic team to familiarize themselves with the innovation and to negotiate their individual interests (“collective action” and “reflexive monitoring” in terms of Normalization Process Theory).

### Preliminary clinical effectiveness

Quantitative findings showed that avoidance behavior, which Radius-Grow was specifically designed to address, remained largely unchanged. Small to medium treatment effects were found on the severity of PTSD symptoms and depressive symptoms in both groups. There were no significant differences between the intervention group and the active control group in terms of any outcome measure. Additionally, the intensity with which the intervention group used Radius-Grow was not related to treatment outcomes. No statistically significant deterioration was observed in either group. Despite its positive feasibility and usability, the software had no additional clinical benefit. Different reasons for these missing effects are possible. Since avoidance behavior did not change in either group, this may be due to a generally low use of in vivo exposure interventions. Due to the naturalistic design, the composition of the face-to-face therapies was not standardized. In several cases, it may have focused e.g. on psychoeducation or stabilization, which are part of trauma-focused Cognitive Behavior Therapy (CBT) [57] . Also, patients used Radius-Grow in different ways, often treating it as a (positive) diary of some kind. Consequently, the match between the digital and face-to-face treatment components may have been imperfect and the way in which Radius-Grow was used may not have aligned with the intended mechanism of reducing avoidance behavior. In line with this, reduction of avoidance behavior was seldom mentioned as a benefit in the qualitative interviews. Therefore, the intervention may have missed its central target mechanism. The added values reported by the participants (e.g., facilitated communication, preparation for therapy sessions and saving positive memories) may not be directly linked to the measured clinical outcomes. Furthermore, it may simply be that the usage intensity was too low to produce any measurable effects.

Previous quantitative studies have shown that digital interventions for PTSD predominantly yield medium to large effects when compared to passive controls (e.g., wait-list) (g = 0.66–0.83), with no additional effects when compared to active controls (e.g., information-only website) [58] . This was in line with the absence of between-group effects in our study, which compared BC to an active control group. Additionally, the overall small to medium positive effect of residential treatment on PTSD symptom severity observed in the current study was consistent with the literature, albeit with smaller effect sizes [59, 60] .

### Limitations

This study captured the initial stage of the implementation process, which has a number of limitations as well as strengths. A lack of standardization regarding the content and intensity of Radius-Grow, and difficulties in understanding its conceptual background, can be seen as the study’s central limitations. These factors impeded successful implementation and the interpretation of (missing) between-group effects. The second major limitation lies in the sequential recruitment protocol. This procedure was deemed necessary to ensure unbiased qualitative data and to prevent the control group from using similar digital tools informally. Consequently, cohort and time effects cannot be distinguished from (missing) between-group effects. Therefore, the between-group comparisons should be interpreted cautiously. In addition to time itself, changes to the clinical staff are a potential confounding variable. This has been mitigated by including the reference clinician as cluster variable in the statistical models. The null correlation between usage intensity and outcomes in the intervention group aligns with the null group differences, underscoring missing intervention effects. Nevertheless, future studies with a quantitative focus should take a randomized recruitment approach, or at least alternate control and intervention cohorts, while assessing possible confounding variables. Regarding the qualitative feedback, there is a potential for a social desirability bias. We have taken measures to mitigate this bias by clearly separating the research and clinical staff. The researcher who conducted the interviews was newly introduced as part of the study procedure and was not involved in any clinical activity. Also, the interview questions were posed open-ended. However, a potential demand characteristic cannot be completely ruled out in an interview study and should be considered in the interpretation of results.

### Strengths

Despite its pitfalls, the naturalistic setting enabled the initial identification of determinants of real-world implementation and the assessment of the feasibility of Radius-Grow in routine residential care setting. This was the study’s primary focus. The use of a multi-method and multi-informant approach (usage data, qualitative interview data, standardized questionnaires, and data from patients and clinicians) provides a coherent and holistic picture within the study’s limited scope.

### Future directions

Future studies in the field should propose “usage profiles” for patients since low usage results in negligible effects while over-usage might be overburdening in the context of a busy residential setting. Instead of aiming at maximizing log-in rates, it may be more important to determine an adequate setting-specific usage intensity. Such usage profiles would have to be embedded into a concise treatment concept, ideally comprising manuals for the face-to-face treatment, the role of digital components and their coordinated use. This concept would have to be accepted and mastered by all participating clinicians to ensure that the core elements of the treatment are conveyed. Within the confines of a manualized therapy, the potential for individualized usage is an advantage, not a flaw, of BC approaches. Future studies should seek a balance between flexibility and clear guidelines for digital tools such that participants can adapt them to their diverse realities and preferences without requiring too much cognitive capacity. In several interviews, establishing cooperation between patients was mentioned as a potential improvement to the BC program. Future versions of Radius-Grow and similar programs might focus on transferring contact between clinicians and patients after discharge into a peer-support network. This would eliminate additional workload and unclear liabilities for clinicians. An example of this is provided by the “a-friend”-app [61] .

## Conclusions

Overall, this study emphasizes the need to consider the multitude of perspectives when implementing health-care interventions and the need for careful training and supervision of professionals. On average, software usability was rated sufficient for implementation. However, clinicians reported added workload, low priority of the digital tool, and difficulties with conceptual integration. Patients reported confusion about expectations and capacity issues. Among both clinicians and patients, some participants seem to have waited for impulses from each other regarding the desired software usage. Sometimes this resulted in a confluence of indecision that led to decreased motivation. In other cases, clinicians and patients’ perspectives aligned, and they used Radius-Grow to collaborate more intensely and to consolidate treatment contents. Regarding quantitative measures of symptom severity, the exploratory data analysis showed no significant added effect of BC over intensive routine care. While the study confirms procedural benefits of BC for the residential treatment of PTSD and its transition into everyday life, uptake may have been insufficient to raise clinical effectiveness. The study therefore underscores the importance of including a setting-specific implementation perspective in the evaluation of BC.

## Supplementary Information

Supplementary Material 1

Supplementary Material 2

## Data Availability

Participant-related data will not be made available in order to guarantee the anonymity of participating patients. Requests to access aggregated data can be directed to Sören Brähmer, soeren.braehmer@uni-bielefeld.de, or Carolin Steuwe, carolin.steuwe@evkb.de.

## Abbreviations

PTSD: Post Traumatic Stress Disorder
BC: Blended Care
CBT: Cognitive Behavior Therapy
TAU: treatment-as-usual
CFIR: Consolidated Framework for Implementation Research
NPT: Normalization Process Theory
