# Supplementary material for: The long road to routine care: piloting the digital mental health intervention for PTSD “Radius Grow” in a psychiatric residential setting
Source: BMC Psychol. 2026 May 15;14:719. doi: 10.1186/s40359-026-04738-5 (PMC13179609; doi:10.1186/s40359-026-04738-5)
Supplement: Supplementary file 1 — Supplementary Material 1 [file 40359_2026_4738_MOESM1_ESM.docx]

**The long road to routine care: piloting the digital mental health intervention for PTSD “Radius Grow” in a psychiatric residential setting**

**Supplemental Material A. Interview guides for semi-structured qualitative interviews.**

Clinician interview guide

1. To what extent is Radius-Grow sensible for your work / the work of your ward?
   1. How useful has Radius-Grow been for your own work so far?
      1. What (expected/unexpected) positive effects of Radius-Grow could you observe?
      2. What (expected/unexpected) negative effects of Radius-Grow could you observe?
   2. What (further) potential added value of Radius-Grow do you see for your work?
   3. How good is the fit of Radius-Grow to the needs of patients?
2. How do you feel about working with Radius-Grow?
   1. Do you think it is your responsibility to use Radius-Grow?
   2. Are there key persons who endorse Radius-Grow and motivate others to work with it?
3. To what extent have you worked with Radius-Grow (as a team)?
   1. How has your work changed due to the use of Radius-Grow? How well does it align with your workflows?
   2. How did the use of Radius-Grow influence the cooperation within the team?
   3. How important was working with Radius-Grow compared to other things?
   4. Do you / your team have the necessary competencies to use Radius-Grow?
   5. Were there enough resources?
   6. How did you get along with the user interface and the different functions?
      1. Were any functions missing or obsolete?
4. To what extent did you reflect on the work with Radius-Grow in the team and give or receive feedback?

Patient interview guide

1. How useful do you find Radius-Grow for your treatment?
   1. What do you think about “Blended Care”, i.e. the combination of digital and personal therapy contents?
2. Please describe the effort you have invested to use Radius-Grow in your treatment.
   1. Did you get acquainted with the functions and the usage of Radius-Grow?
   2. How difficult is it, to learn the handling of Radius-Grow?
      1. How much/what kind of support did you need to use it?
      2. Did you have problems while using Radius-Grow?
      3. What would have facilitated the usage?
   3. To what extent have you used Radius-Grow?
3. How did Radius-Grow affect your treatment?
   1. What positive aspects/effects were there?
   2. What negative aspects/effects were there?
   3. What was your experience with the “Collecting moments” function?
      1. Have you used the “Collect moments” function?
      2. Did you find collecting moments on the tree rewarding?
      3. Did Radius-Grow help you to reduce avoidance behavior?
   4. Which functions were particularly important/useful for you?
   5. Which functions were less important to you?
   6. Which functions did you miss?
4. How satisfied are you with Radius-Grow?
   1. Was Radius-Grow adequately tailored to your needs?
   2. Do you find the user interface appealing?
   3. How do you rate the menu navigation, loading times, design, etc.?
   4. Do you trust the data security of Radius-Grow?
   5. Will you continue to work with Radius-Grow after discharge?
5. To what extent did you reflect on Radius-Grow with others?
   1. Who did you talk to and what did you talk about?
   2. Have you given or received feedback on Radius-Grow?

Note: detailed questions in both interview guides (labelled with characters and roman numerals) were only posed when necessary.
