# Supplementary material for: The long road to routine care: piloting the digital mental health intervention for PTSD “Radius Grow” in a psychiatric residential setting
Source: BMC Psychol. 2026 May 15;14:719. doi: 10.1186/s40359-026-04738-5 (PMC13179609; doi:10.1186/s40359-026-04738-5)
Supplement: Supplementary file 2 — Supplementary Material 2 [file 40359_2026_4738_MOESM2_ESM.docx]

**The long road to routine care: piloting the digital mental health intervention for PTSD “Radius Grow” in a psychiatric residential setting**

**Supplemental Material B. Results from multilevel models.**

Table S1. Estimated means and effect sizes based on estimated means from questionnaire data.

| **Construct**  **(Measure)** | **Group** | **Measurement** | | |
| --- | --- | --- | --- | --- |
|  |  | Admission  [m^1^ (se^2^)  g_within_^3^  g_between_^3^] | Discharge  [m^1^ (se^2^)  g_within_^3^  g_between_^3^] | 3-m. follow-up  [m^1^ (se^2^)  g_within_^3^  g_between_^3^] |
| PTSD  Symptoms  (ITQ^4^) | Intervention | 19.59 (1.12)  g_within_=n.a. | 16.53 (1.13)  g_within_=-0.65 | 16.52 (1.28)  g_within_=-0.64 |
|  | Control | 19.47 (1.24)  g_within_=n.a.  g_between_=-0.03 | 17.01 (1.30)  g_within_=-0.51  g_between_=0.11 | 17.24 (1.38)  g_within_=-0.46  g_between_=0.16 |
| Avoidance behavior  (PABQ^5^) | Intervention | 68.76 (3.64)  g_within_=n.a. | 65.72 (3.69)  g_within_=-0.21 | 72.01 (4.21)  g_within_=0.22 |
|  | Control | 62.10 (4.10)  g_within_=n.a.  g_between_=-0.48 | 62.43 (4.29)  g_within_=0.02  g_between_=-0.24 | 66.39 (4.50)  g_within_=0.28  g_between_=-0.41 |
| Depressive symptoms  (BDI-V^6^) | Intervention | 71.38 (4.88)  g_within_=n.a. | 59.96 (4.95)  g_within_=-0.58 | 65.12 (5.62)  g_within_=-0.31 |
|  | Control | 67.68 (5.52)  g_within_=n.a.  g_between_=-0.20 | 58.65 (5.76)  g_within_=-0.45  g_between_=-0.07 | 63.11 (6.04)  g_within_=-0.22  g_between_=-0.11 |
| Patient-rated working alliance  (WAI-SR-P^7^) | Intervention | 4.08 (0.16)  g_within_=n.a. | 4.37 (0.17)  g_within_=0.43 | - |
|  | Control | 3.96 (0.18)  g_within_=n.a.  g_between_=-0.18 | 3.94 (0.19)  g_within_=-0.03  g_between_=-0.67 | - |
| Clinician-rated working alliance  (WAI-SR-T^8^) | Intervention | 3.83 (0.14)  g_vwithin_=n.a. | 3.97 (0.14)  g_within_=0.26 | - |
|  | Control | 3.85 (0.16)  g_within_=n.a.  g_between_=0.03 | 4.05 (0.17)  g_within_=0.37  g_between_=0.14 | - |

^1^Estimated mean from linear mixed model ^2^Standard error of the mean ^3^Hedges’ g: within-group effect-sizes compare discharge and follow-up values to admission values; between-group effect-sizes compare each measurement in the control group to the corresponding measurement in the intervention group; g>0.2 small effect, g>0.5 medium effect, g>0.8 large effect ^4^International Trauma Questionnaire ^5^Posttraumatic Avoidance Behavior Questionnaire ^6^Beck Depression Inventory V ^7^Working Alliance Inventory-Short-Revised-Patient Version ^8^Working Alliance Inventory-Short-Revised-Therapist Version

Table S2. ANOVA for linear mixed model predicting PTSD symptoms from group^1^.

| **Predictor** | **F-value** | **Numerator DF**^2^ | **Denominator DF**^2^ | ***P* (>F)** |
| --- | --- | --- | --- | --- |
| Measurement | 6.65 | 2 | 56.05 | .003 |
| Group | 0.08 | 1 | 35.08 | .779 |
| Measurement*Group | 0.12 | 2 | 56.05 | .888 |

^1^Model formula: ITQ ~ 1 + Measurement*Group + (1 | Clinician / Patient).

^2^DF: Degrees of Freedom.

Table S3. ANOVA for linear mixed model predicting avoidance behavior from group^1^.

| **Predictor** | **F-value** | **Numerator DF**^2^ | **Denominator DF**^2^ | ***P* (>F)** |
| --- | --- | --- | --- | --- |
| Measurement | 1.39 | 2 | 57.63 | .257 |
| Group | 1.60 | 1 | 25.24 | .217 |
| Measurement*Group | 0.18 | 2 | 57.62 | .833 |

^1^Model formula: PABQ ~ 1 + Measurement*Group + (1 | Clinician / Patient).

^2^DF: Degrees of Freedom.

Table S4. ANOVA for linear mixed model predicting depressive symptoms from group^1^.

| **Predictor** | **F-value** | **Numerator DF**^2^ | **Denominator DF**^2^ | ***P* (>F)** |
| --- | --- | --- | --- | --- |
| Measurement | 3.87 | 2 | 56.13 | .027 |
| Group | 0.17 | 1 | 22.61 | .682 |
| Measurement*Group | 0.06 | 2 | 56.10 | .946 |

^1^Model formula: BDI ~ 1 + Measurement*Group + (1 | Clinician / Patient).

^2^DF: Degrees of Freedom.

Table S5. ANOVA for linear mixed model predicting patient-rated working alliance from group^1^.

| **Predictor** | **F-value** | **Numerator DF**^2^ | **Denominator DF**^2^ | ***P* (>F)** |
| --- | --- | --- | --- | --- |
| Measurement | 1.39 | 1 | 29.51 | .249 |
| Group | 1.93 | 1 | 33.76 | .174 |
| Measurement*Group | 1.92 | 1 | 29.51 | .177 |

^1^Model formula: WAI-SR-P ~ 1 + Measurement*Group + (1 | Clinician / Patient).

^2^DF: Degrees of Freedom.

Table S6. ANOVA for linear mixed model predicting clinician-rated working alliance from group^1^.

| **Predictor** | **F-value** | **Numerator DF**^2^ | **Denominator DF**^2^ | ***P* (>F)** |
| --- | --- | --- | --- | --- |
| Measurement | 3.50 | 1 | 29.47 | .071 |
| Group | 0.09 | 1 | 30.49 | .769 |
| Measurement*Group | 0.12 | 1 | 29.34 | .736 |

^1^Model formula: WAI-SR-T ~ 1 + Measurement*Group + (1 | Clinician / Patient).

^2^DF: Degrees of Freedom.

Table S7. Standardized LMM regression coefficients of Login rate (predicting symptom change)^1^.

| **Outcome** | **Standardized regression coefficient of Login rate**  β [95%-CI] | |
| --- | --- | --- |
|  | **Discharge** | **Follow-up** |
| PTSD symptom change | .12 [-.44, .68] | .08 [-.54, .70] |
| Avoidance behavior change | .10 [-.41, .60] | .04 [-.52, .60] |
| Depressive symptom change | <.001 [-.54, .53] | .36 [-.24, .95] |
| Patient-rated working alliance change | .02 [-.31, .35] | - |
| Clinician-rated working alliance change | -.01 [-.34, .31] | - |

^1^Model specifications see tables 8-12.

Table S8. ANOVA for linear mixed model predicting PTSD symptom change from login rate^1^.

| **Predictor** | **F-value** | **Numerator DF**^2^ | **Denominator DF**^2^ | ***P* (>F)** |
| --- | --- | --- | --- | --- |
| Measurement | 0.00 | 1 | 21.91 | .995 |
| Login rate | 0.21 | 1 | 21.97 | .649 |
| Admission score | 2.04 | 1 | 16.01 | .172 |
| Measurement*Login rate | 0.01 | 1 | 21.34 | .917 |

^1^Model formula: ITQ change score ~ 1 + Measurement*Login rate + Admission score + (1 | Clinician / Patient).

^2^DF: Degrees of Freedom.

Table S9. ANOVA for linear mixed model predicting avoidance behavior change from login rate^1^.

| **Predictor** | **F-value** | **Numerator DF**^2^ | **Denominator DF**^2^ | ***P* (>F)** |
| --- | --- | --- | --- | --- |
| Measurement | 0.70 | 1 | 22.08 | .411 |
| Login rate | 0.13 | 1 | 24.96 | .721 |
| Admission score | 4.35 | 1 | 16.35 | .053 |
| Measurement*Login rate | 0.03 | 1 | 21.51 | .874 |

^1^Model formula: PABQ change score ~ 1 + Measurement*Login rate + Admission score + (1 | Clinician / Patient).

^2^DF: Degrees of Freedom.

Table S10. ANOVA for linear mixed model predicting depressive symptom change from login rate^1^.

| **Predictor** | **F-value** | **Numerator DF**^2^ | **Denominator DF**^2^ | ***P* (>F)** |
| --- | --- | --- | --- | --- |
| Measurement | 0.32 | 1 | 21.33 | .579 |
| Login rate | 0.79 | 1 | 24.75 | .381 |
| Admission score | 0.13 | 1 | 22.13 | .718 |
| Measurement*Login rate | 0.92 | 1 | 20.79 | .350 |

^1^Model formula: BDI change score ~ 1 + Measurement*Login rate + Admission score + (1 | Clinician / Patient).

^2^DF: Degrees of Freedom.

Table S11. ANOVA for linear mixed model predicting patient-rated working alliance change from login rate^1^.

| **Predictor** | **F-value** | **Numerator DF**^2^ | **Denominator DF**^2^ | ***P* (>F)** |
| --- | --- | --- | --- | --- |
| Login rate | 0.01 | 1 | 14.83 | .913 |
| Admission score | 0.003 | 1 | 13.70 | .955 |

^1^Model formula: WAI-SR-P change score ~ 1 + Login rate + Admission score + (1|Clinician).

Since the working alliance was measured only at admission and at discharge, only one change score was computed. Therefore, measurement was not included as predictor. Since only one observation per patient remained, nesting of observations in patients was obsolete. Observations were still nested in clinicians.

^2^DF: Degrees of Freedom.

Table S12. ANOVA for linear mixed model predicting clinician-rated working alliance change from login rate^1^.

| **Predictor** | **F-value** | **Numerator DF**^2^ | **Denominator DF**^2^ | ***P* (>F)** |
| --- | --- | --- | --- | --- |
| Login rate | 0.01 | 1 | 26.93 | .930 |
| Admission score | 14.89 | 1 | 19.34 | .001 |

^1^Model formula: WAI-SR-T change score ~ 1 + Login rate + Admission score + (1|Clinician).

Since the working alliance was measured only at admission and at discharge, only one change score was computed. Therefore, measurement was not included as predictor. Since only one observation per patient remained, nesting of observations in patients was obsolete. Observations were still nested in clinicians.

^2^DF: Degrees of Freedom.
